# Genetic Structure in the Coral, *Montastraea cavernosa*: Assessing Genetic Differentiation among and within Mesophotic Reefs

**DOI:** 10.1371/journal.pone.0065845

**Published:** 2013-05-29

**Authors:** Daniel A. Brazeau, Michael P. Lesser, Marc Slattery

**Affiliations:** 1 Department of Pharmaceutical Sciences, University of New England, Portland, Maine, United States of America; 2 Department of Molecular Cellular and Biomedical Sciences, University of New Hampshire, Durham, New Hampshire, United States of America; 3 Department of Pharmacognosy, University of Mississippi, University, Mississippi, United States of America; University of Texas, United States of America

## Abstract

Mesophotic coral reefs (30–150 m) have recently received increased attention as a potential source of larvae (e.g., the refugia hypothesis) to repopulate a select subset of the shallow water (<30 m) coral fauna. To test the refugia hypothesis we used highly polymorphic Amplified Fragment Length Polymorphism (AFLP) markers as a means to assess small-scale genetic heterogeneity between geographic locations and across depth clines in the Caribbean coral, *Montastraea cavernosa*. Zooxanthellae-free DNA extracts of coral samples (N = 105) were analyzed from four depths, shallow (3–10 m), medium (15–25 m), deep (30–50 m) and very deep (60–90 m) from Little Cayman Island (LCI), Lee Stocking Island (LSI), Bahamas and San Salvador (SS), Bahamas which range in distance from 170 to 1,600 km apart. Using AMOVA analysis there were significant differences in Φ_ST_ values in pair wise comparisons between LCI and LSI. Among depths at LCI, there was significant genetic differentiation between shallow and medium versus deep and very deep depths in contrast there were no significant differences in Φ_ST_ values among depths at LSI. The assignment program AFLPOP, however, correctly assigned 95.7% of the LCI and LSI samples to the depths from which they were collected, differentiating among populations as little as 10 to 20 m in depth from one another. Discriminant function analysis of the data showed significant differentiation among samples when categorized by collection site as well as collection depth. F_ST_ outlier analyses identified 2 loci under positive selection and 3 under balancing selection at LCI. At LSI 2 loci were identified, both showing balancing selection. This data shows that adult populations of *M. cavernosa* separated by depths of tens of meters exhibits significant genetic structure, indicative of low population connectivity among and within sites and are not supplying successful recruits to adjacent coral reefs less than 30 m in depth.

## Introduction

Given the widespread damage common to most coral reefs [1], [2], [3], coral reef ecosystems are becoming fragmented and disconnected in an ecological context. This is particularly true for Caribbean reef systems [4] where increasing rates of anthropogenic disturbance, including climate change, suggest that resilience is low and significant recovery of most reefs is unlikely [5], [6]. Marine protected areas (MPA) have been proposed as one of the principle means to attempt to conserve threatened marine ecosystems in general and coral reefs in particular. Globally over 5,000 MPAs covering more than 3 million sq. km have been designated (United Nations Environment Programs World Conservation Monitoring Center, marine protected database www.wdpa-marine.org). The effective design and management of MPAs requires an understanding of the nature and extent of the movement of individuals and their successful establishment within and among protected areas. While most assessments of the genetic connectivity between populations of corals are conducted on reefs leass than 30 m in depth [7], [8], the role that deep, mesophotic, populations play as potential sources of successful recruits to shallower more disturbed reefs is largely unknown [9], [10].

Using genetic data we can gain important insight into overall population connectivity [11] among shallow and deep mesophotic populations of corals. Population connectivity includes not only the movement of individuals into and out of populations, but also the relative importance of these recruits to the intrinsic growth rates of each population. Assessing the extent of differences in allele frequencies among populations provides an estimate of the level of connectedness that includes the action of local selection which determines in part the pool of adults available for reproduction [12].

An assessment of gene flow among populations has traditionally employed population genetic methodologies based upon comparisons of allele frequency distributions among populations to estimate classic parameters like *Nm* and *F_ST_*. These indirect genetic indices of connectivity have been problematic [11], [13], [14], [15], in that such estimates are based upon simple and generally unrealistic equilibrium population models. More recently, with the availability of highly variable and abundant genetic markers, it is now possible to identify and assign individuals to populations [16], [17]. Direct estimates of genetic connectivity through the genetic assignment of individuals to source populations (with some probability) are in many ways similar to studies employing chemical or environmental signatures to assess the origin of individuals [18]. These methodologies can potentially be used to not only assess the relative contribution of immigration to a population, but with adequate sampling, the source population of immigrants. Further, genetic markers like microsatellites, single nucleotide polymorphisms (SNPs) and AFLPs that simultaneously assay numerous loci within the genome, allows for a more precise view of the ecological (immigration, emigration) and evolutionary factors (parentage analysis, hybridization, selection) that affect the survival of populations.

AFLPs have been used widely in plants, bacteria, and fungi though AFLPs have been underutilized as a population genetic marker in animals [19]. AFLPs have been used successfully to determine migration rates [20], species boundaries [21], parental contributions to populations [22] and in the analysis of quantitative traits [23]. AFLPs provide an economical, potentially genome-wide assessment (i.e., a genomic fingerprint) of genetic similarity and are highly reproducible [24], [25]. AFLPs are well suited for population assignment studies [26], [27], [28], [29] where the number of polymorphic loci is more important than allelic diversity [30]. In addition the protocols for AFLPs are nearly universal for most species and can be rapidly adapted to corals and other members of the coral reef community. It should be noted that recent massively parallel sequencing technologies may provide the same advantages [31] though the cost is likely to be higher for the immediate future. There is increasing interest in the use of AFLPs on corals and other symbiotic cnidarians [32] and AFLPs along with other genetic markers have been used to assess the nature of species boundaries in the *Montastraea annularis* complex [33] and population connectivity and assignment in a number of scleractinian corals [34], [35], [36].

AFLPs also have limitations; as dominant markers, each band has at maximum half the information content compared to microsatellites [37] or SNPs. More problematic, it is impossible to directly assess departures from Hardy-Weinberg equilibrium with dominant markers. Additionally, most studies (including this one) assume homology among bands in the same size “bin”. Homoplasy is likely minimized as bin sizes become smaller (here we used 5 bp increments) and when comparisons are among the same taxonomic group [38], [39] as we have done in this study. Finally, in that the genomic location of AFLPs markers are unknown the independence of each marker as a measure of population differentiation should be tested. We did not find any associations among the markers used in this study however, our small sample sizes restricts our ability to detect all but nearly perfect linkage. Here, we use AFLPs to assess the genetic structure among populations from shallow and mesophotic depths, and between three Caribbean populations of the scleractinian coral, *Montastraea cavernosa.*

## Results

A total of 105 coral samples were analyzed from three locations, LCI, LSI, Bahamas and SS. For the analyses described below coral samples were binned into shallow (3–10 m), medium (15–25 m), deep (30–50 m) and very deep (60–90 m) categories for all locations. The binning was done prior to analysis in order to minimize sample size differences among groups (Table 1). Samples were analyzed using three AFLP selective primer sets yielding 86 polymorphic markers with band frequencies ranging as high as 67%. The average band frequency per individual was 25%. Repeatability of individual band assignment (either present or absent) among the three replicate runs for each primer set was very high. Overall 91.6% of the band assignments following 1 run of all samples were unchanged following the second run (replicate). Similarly 92.8% of the assignments were identical following the third run (Table 2). We have undertaken a number of precautions to minimize genotyping and experimental error that may be incurred in the AFLP assay. Specifically, for all steps in the assay including DNA isolation, restriction-ligation, pre-selective PCR, selective PCR, electrophoresis and band scoring the samples were processed in large batches that purposefully included samples from all populations to be assayed. In this way any experimental artifacts that could arise due to temporal variation in reaction conditions would not bias the analysis. In addition, all reaction components (buffers, dNTP's, polymerase, primers) were drawn from a single stock in order to minimize variance in lots. Finally, to avoid unknown artifacts that may result in differences in marker diversity among sites [40] band assignment was automated (GeneMarker®, SoftGenetics LLC State College, PA) using the same criteria for all runs as has been suggested elsewhere [25].

**Table 1:** Sample sizes at each depth at each of the three collection sites.

		Depth (m)	LCI	LSI	SS	Total
Shallow	S	3–10	7	15	2	24
Medium	M	15–25	11	11	2	24
Deep	D	30–50	18	11	5	34
Very Deep	VD	60–90	5	9	9	23
Total			41	46	18	105

LCI – Little Cayman Island, LSI – Lee Stocking Island, SS – San Salvador. Depth categories were chosen prior to analyses with the goal of creating groups of equal sample size.

**Table 2:** Genotyping statistics for each AFLP selective primer pair.

Primer Pairs	EAMA	EAMB	EBMA	Total
Number of polymorphic markers	27	29	30	86
Total Number of markers scored (+ or −)	2,511	2,697	2,790	7,998
Number scored present (+) in 1 of 3 replicates	973	1,122	1,394	3,489
Number scored present (+) in 2 of 3 replicates	804	914	1,103	2,821
Number scored present (+) in 3 of 3 replicates	661	738	845	2,244
Percent of markers unchanged from 1 to 2 replicates	93.3%	92.3%	89.6%	91.6%
Percent of markers unchanged from 2 to 3 replicates	94.3%	93.5%	90.8%	92.8%

The nested AMOVA analyses revealed significant genetic heterogeneity in comparisons between the eight sampling depths from the LCI and LSI with an overall average Φ_ST_ of 0.088. Samples from SS were not included in this AMOVA analyses due to small sample sizes. The component of variance between groups (LCI and LSI) was 7.8% compared to 1.0% among samples within groups (Table 3). Bootstrapped pair wise comparisons between the sampled depths at LCI and LSI showed significant differences among shallow, medium and deep comparisons, but no differentiation between the two very deep depths (Table 3). Analyses of the four depths within LCI exhibited no significant differences between the shallow and medium or between the deep and very deep depths, however there were significant genetic differences among comparisons between shallow and medium depths versus the deep and very deep depths (Table 3). In contrast, the populations sampled at LSI showed no similar significant differences in Φ_ST_ values.

**Table 3:** Pairwise comparisions of genetic differentiation between depths sampled at Little Cayman Island and Lee Stocking Island using a nested Analysis of Molecular Variance (AMOVA).

Nested Analysis		df	Variance components	Percent of variation
Variance among Groups (LCI and LSI)		1	1.373	7.8%
Variance among populations within groups		6	0.168	1.0%
Variance within populations		79	15.996	91.2%
Total		86	17.537	

		Little Cayman Island				Lee Stocking Island			
		Shallow 3–10 m	Medium 15–25 m	Deep 30–50 m	Very Deep 60–90 m	Shallow 3–10 m	Medium 15–25 m	Deep 30–50 m	Very Deep 60–90 m
	Shallow	–	0.7802	0	0.014	0			
LCI	Med	0	–	0	0		0		
	Deep	**0.0695**	**0.0594**	–	0.3966			0	
	Very Deep	**0.1066**	**0.1096**	0.0327	–				0.6124
	Shallow	**0.0762**				–	0.3716	0.5215	0.4505
LSI	Med		**0.1731**			0	–	0.6444	0.5005
	Deep			**0.0889**		0	0	–	0.6613
	Very Deep				0	0	0	0	–

Significance levels based upon bootstrap analysis for 5000 iterations (upper diagonal). Φ_ST_ values shown on lower diagonal. Significant Φ_ST_ values indicated in bold.

The AFLPOP analysis of the randomized data set did not reveal any structure that might arise as an artifact given the large number of markers and small population sizes. There were few cases of self-assignment (7 individuals out of 87 in the analysis), that is, the assignment of an individual back to the population from which it was “collected” (Table 4). In contrast the AFLPOP analyses of the same data set without randomization revealed significant population structure among sampled depths from LSI and LCI (Table 5A&B). Overall 95.7% of the 87 individual samples could be correctly assigned back to the population from which they were collected (Table 5A). At the higher assignment threshold, 85.6% of the samples were correctly assigned to the population from which they were collected (Table 5B). Only 13.5% of the samples could not be assigned to any single population.

**Table 4:** Assignment of **randomized** samples based upon AFLPOP analysis of band frequencies.

		Little Cayman Island				Lee Stocking Island			
	allocated to:	Shallow 3–10 m	Medium 15–25 m	Deep 30–50 m	Very Deep 60–90 m	Shallow 3–10 m	Medium 15–25 m	Deep 30–50 m	Very Deep 60–90 m
Little Cayman Island	Shallow								
	Medium			11.1%		6.7%	9.1%	18.2%	22.2%
	Deep	28.6%	9.1%		40.0%	20.0%	9.1%		
	Very Deep	14.3%	9.1%	16.7%		13.3%	18.2%	18.2%	11.1%
Lee Stocking Island	Shallow	14.3%	18.2%	22.2%	60.0%	**13.3%**	18.2%	27.3%	22.2%
	Medium	14.3%	9.1%	5.6%			**9.1%**		11.1%
	Deep	14.3%	18.2%	11.1%		33.3%	27.3%	**36.4%**	33.3%
	Very Deep	14.3%	36.4%	33.3%		13.3%	9.1%		

Assignments based upon 500 simulations of the randomized dataset. Assignments with log likelihood threshold set to 0 in the analysis. Samples were assigned to site with the highest likelihood. Proportions of individuals correctly assigned to the “source” population are in bold on the diagonal.

**Table 5:** Assignment of samples based upon AFLPOP analysis of band frequencies at each collection depth.

A) Samples assigned to depths with the highest likelihood.									
		Little Cayman Island				Lee Stocking Island			
	allocated to:	Shallow 3–10 m	Medium 15–25 m	Deep 30–50 m	Very Deep 60–90 m	Shallow 3–10 m	Medium 15–25 m	Deep 30–50 m	Very Deep 60–90 m
Little Cayman Island	Shallow	**89.8%**	2.4%	5.4%	1.8%		0.2%	0.8%	
	Medium	2.0%	**93.2%**	3.2%	0.8%				
	Deep	4.4%	2.0%	**89.6%**				0.4%	
	Very Deep	1.2%	2.4%	1.4%	**97.0%**				
Lee Stocking Island	Shallow					**99.4%**	1.8%		
	Medium					0.6%	**98.0%**		
	Deep	2.4%			0.2%			**98.8%**	
	Very Deep	0.2%		0.4%	0.2%				**100.0%**

B) Assignments with log likelihood threshold set to 0									
		Little Cayman Island				Lee Stocking Island			
	allocated to:	Shallow 3–10 m	Medium 15–25 m	Deep 30–50 m	Very Deep 60–90 m	Shallow 3–10 m	Medium 15–25 m	Deep 30–50 m	Very Deep 60–90 m
Little Cayman Island	Shallow	**66.4%**	0.2%	1.4%	0.2%			0.2%	
	Medium	0.6%	**79.8%**	0.6%					
	Deep	0.6%	0.4%	**69.0%**					
	Very Deep	0.6%		0.4%	**89.8%**				
Lee Stocking Island	Shallow					**95.8%**	1.0%		
	Medium					0.2%	**92.4%**	0.2%	
	Deep	0.2%						**92.8%**	
	Very Deep	0.0%	0.2%	0.2%					**98.8%**
	None	31.6%	19.4%	28.4%	10.0%	4.0%	6.6%	6.8%	1.2%

Assignments percentages based upon 500 simulations. A) Samples assigned to depths with the highest likelihood. B) Assignments with log likelihood threshold set to 0. Samples assigned to a depth only if the next most likely assignment is 10 fold less. Samples assigned to “None” could not be assigned to a single depth with a probability 10 fold greater than any other depth. Proportions of individuals “correctly” assigned back to population from which they were collected are on the diagonal.

To assess genetic similarities potentially due to depth we repeated the AFLPOP analysis of the entire data set (LCI, LSI and SS combined) categorized by depth alone. There was significant similarity among the samples (Table 6A&B) as measured by the ability to correctly assign individuals samples back to the correct collection depths regardless of geographic location. An overall average of 79.5% of the samples were correctly assigned back to the depth categories from which they were collected. At the more stringent assignment threshold of “1” 42.6% of the samples were assigned to the depth from which they were collected with a probability 10 times greater than the next most likely population. In both analyses, samples from the deep and very deep depths had the highest self-assignment frequencies suggesting these samples were the most genetically distinct.

**Table 6:** Assignment based upon AFLPOP analysis of band frequencies of samples categorized only by depth (LCI, LSI and SS collection sites combined).

A) Samples assigned to depth with the highest likelihood				
Allocated to:	Shallow 3–10 m	Medium 15–25 m	Deep 30–50 m	Very Deep 60–90 m
Shallow	**73.2%**	13.3%	8.0%	4.2%
Medium	11.9%	**75.4%**	6.8%	3.0%
Deep	8.9%	6.6%	**80.2%**	3.6%
Very Deep	6.0%	4.7%	5.0%	**89.3%**

B) Assignments with log likelihood threshold set to 0				
Allocated to:	Shallow 3–10 m	Medium 15–25 m	Deep 30–50 m	Very Deep 60–90 m
Shallow	**27.6%**	0.8%	0.7%	0.3%
Medium	1.1%	**34.6%**	0.4%	0.2%
Deep	0.9%	0.7%	**44.6%**	0.3%
Very Deep	1.0%	0.9%	0.8%	**63.6%**
None	69.5%	63.1%	53.6%	35.7%

Assignments percentages based upon 500 simulations. Samples assigned to depth only if the next most likely assignment is 10 fold less. Samples assigned to “None” could not be assigned to a single depth with a probability 10 fold greater than any other depth. Proportions of individuals “correctly” assigned back to population from which they were collected are on the diagonal.

**Figure 1:**
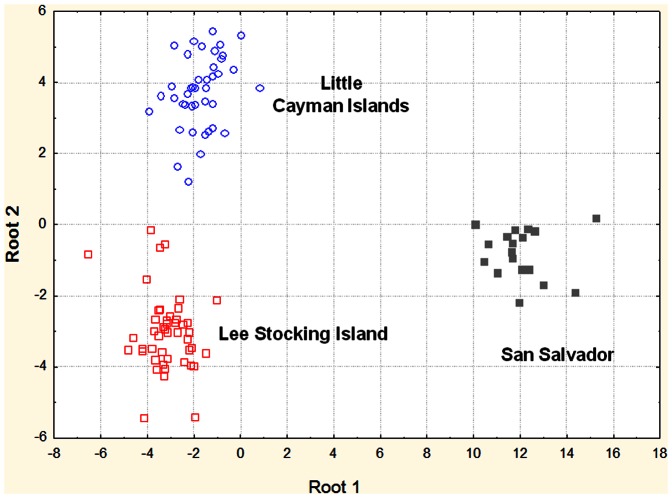
Plot of standardized coefficients for canonical variables based upon discriminant function analysis of samples classified only by collection sites. Little Cayman Island –open circles, San Salvador – closed squares, Lee Stocking Island – open squares.

**Figure 2:**
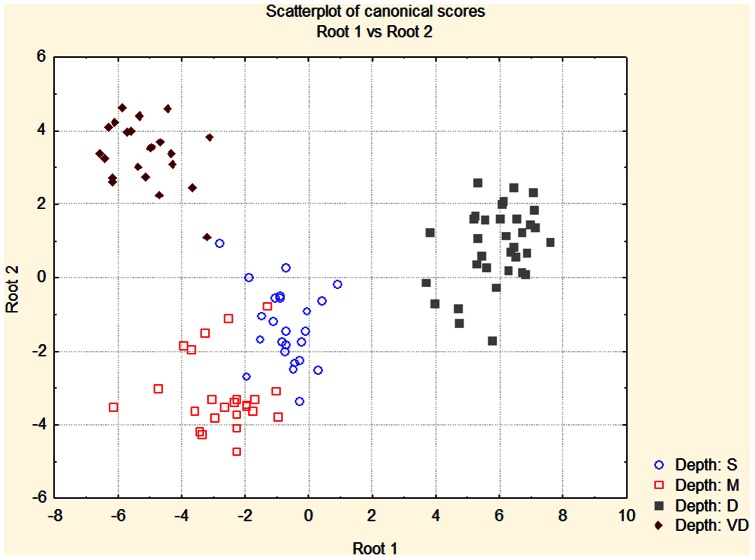
Plot of standardized coefficients for canonical variables based upon discriminant function analysis of samples classified only by collection depth. Shallow (3–10 m) - circles, Medium depth (15–25 m) – open squares, Deep (30–50 m) – closed squares, and Very Deep (60–90) – diamonds

For the Discriminant Function Analysis (DFA) a step-wise analysis method was used to build a model using the markers that contributed significantly to the discriminant function. All markers were evaluated and added one at a time to the model beginning with the marker that contributed most to the discrimination between groups. Additional variables were added until the respective F-values for the variable were >1.0. In order to avoid the addition of redundant markers to the model, markers with tolerance-values (calculated as 1-R^2^ of the variable, compared with all other variables in the model) <0.01 were excluded. The DFA of the randomized data set failed to identify any markers that would significantly differentiate among groups. In the first analysis using the actual data set, 16 of the 86 markers were necessary to build a model that significantly differentiated among the three populations (LCI, LSI and SS) without respect to depth (Table 7). ANOVA analysis of each of the markers used in the models indicated that all but two were significantly different among populations. A scatter plot of the canonical scores for each individual shows clear discrimination of all populations (Fig. 1). In a second analysis to assess the ability to discriminate among samples solely based upon depth 8 of the 86 markers were necessary (Table 8). A plot of the significant canonical scores shows clear distinction among depths with some overlap between samples from the shallow and medium depths (Fig. 2). Significant discrimination was possible even though samples from the three sampling locations were combined.

**Table 7:** Band frequencies for AFLP markers used in discriminant function analysis (DFA).

	Marker ID	Little Cayman Island	Lee Stocking Island	San Salvador	All Groups	ANOVA p
		41	46	18	105	
1	129	0.098	0.630	0.222	0.352	0.000
2	138	0.098	0.370	0.000	0.200	0.000
3	199	0.268	0.565	0.833	0.495	0.000
4	128	0.171	0.478	0.333	0.333	0.009
5	50	0.415	0.304	0.111	0.314	0.068
6	59	0.561	0.761	0.500	0.638	0.063
7	55	0.293	0.609	0.167	0.410	0.001
8	208	0.293	0.370	0.000	0.276	0.011
9	211	0.000	0.217	0.000	0.095	0.001
10	206	0.146	0.217	0.000	0.152	0.094
11	212	0.293	0.304	0.000	0.248	0.027
12	196	0.366	0.609	0.167	0.438	0.002
13	195	0.561	0.565	0.333	0.524	0.210
14	121	0.390	0.457	0.056	0.362	0.009
15	70	0.268	0.217	0.056	0.210	0.182
16	133	0.366	0.630	0.722	0.543	0.011

In this analysis samples were identified only by collection site. Markers listed in order of their contribution to discrimination of collection sites (LCI, LSI, and SS).

**Table 8:** Band frequencies for AFLP markers used in discriminant function analysis (DFA).

DFA			Marker Frequencies				
	Marker ID	ANOVA p value	Shallow	Medium	Deep	Very Deep	All
			24	24	34	23	105
1	67	0.005	0.38	0.29	0.15	0.00	0.20
2	185	0.006	0.08	0.04	0.26	0.00	0.11
3	198	0.041	0.42	0.54	0.44	0.78	0.53
4	47	0.088	0.17	0.33	0.21	0.04	0.19
5	51	0.177	0.17	0.13	0.35	0.22	0.23
6	68	0.095	0.13	0.29	0.06	0.13	0.14
7	135	0.075	0.08	0.33	0.18	0.09	0.17
8	45	0.013	0.08	0.21	0.38	0.09	0.21

In this analysis samples were identified only by depth. Markers listed in order of their contribution to discrimination of depth categories.

**Figure 3:**
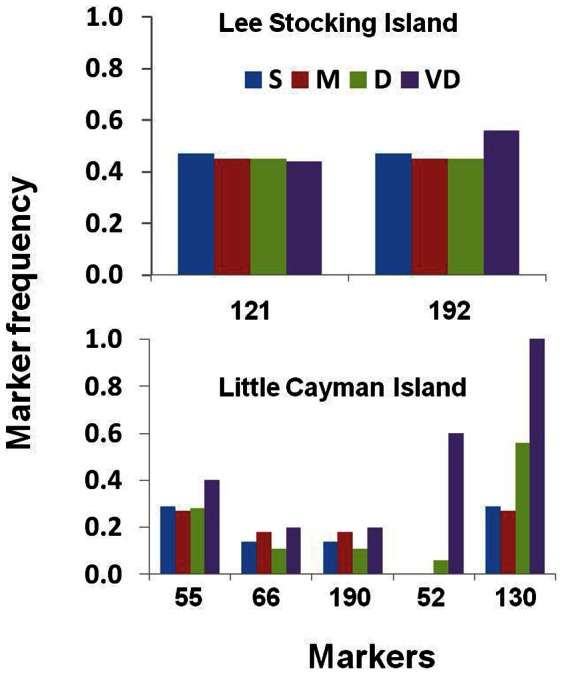
Plot of marker frequencies for three markers identified in the F_ST_ outlier analysis to exhibit significant deviation from neutral expectations. The three markers in the Lee Stocking Island samples all showed significantly higher F_ST_ compared to neutral expectations suggesting positive selection. The three markers in the Little Cayman Island data set all showed significantly lower F_ST_ suggesting balancing selection.

F_ST_ outlier analysis of the 86 AFLP markers identified 7 markers for the LCI and LSI sites that may be affected by selection. For each analysis putative outlier markers were initially identified and removed for the computation of an unbiased distribution of neutral F_ST_s. The second and final run for each data set included all markers to evaluate each marker F_ST_ compared to neutral expectations. At LCI 2 markers were identified that displayed significantly greater differentiation than expected suggesting positive selection and 3 markers that displayed significantly less differentiation than expected suggesting balancing selection (Fig. 3). At LSI 2 markers were identified that displayed significantly less differentiation than expected, again suggesting balancing selection (Fig. 3). None of the outlier loci were identified in the DFA analysis. The AFLPOP analysis repeated excluding the outlier loci did not change the rates of self assignment. At LCI rates ranged from 97% to 99% compared with 89.6% to 97% with all loci, at LSI rates of self assignment without the outlier loci in the analysis ranged from 87% to 96% compared to 98% to 100%. Not surprisingly the rates improved slightly at LCI where all 3 outlier loci were attributed to balancing selection.

## Discussion

As have a number of recent studies examining genetic structure among broadcast spawning corals [7], [41], [42] we found significant genetic differentiation among the populations of a broadcast spawner with presumably high dispersal abilities. Our findings would seem to be at odds with Nunes *et al.* [43] that recently found no significant genetic differences among 191 *M. cavernosa* individuals sampled from eight populations separated by as much as 3000 km. This study [43] employed data from three loci (one mitochondrial and two nuclear) encompassing 2,407 bp of sequence. Here, using 86 polymorphic markers we were able to detect clear genetic differences among populations on a much smaller scale; between LCI and LSI (a distance of less than 1500 km) and among depths at LCI (less than a km in total extent). The differences observed are likely due to the greater number of independent markers used in this study some of which may be under the influence of selection. Clearly, and beyond the scope of this study, local and regional oceanography will influence the dispersal of larvae and therefore the genetic structure of coral populations [8], [44], but our detection of genetic structure clearly suggests factors other than dispersal alone play a role in genetic makeup of populations. Finally, Nunes *et al.* [45] and another study [46] have shown no evidence for depth specific cryptic species within *M. cavernosa* and thus do not explain the genetic patterns observed here.

Significant genetic differences were detected using the indirect genetic measure, Φ_ST_, and the direct assignment-based techniques, AFLPOP. Interestingly, AFLPOP was able to detect genetic structure among the sampled populations at LSI where we found no significant differences in the Φ_ST_ values. This disagreement between the two methods may reflect the sensitivity of indirect genetic estimates of population structure to the small sample sizes available here and the underlying assumptions of the models upon which Φ_ST_ values are based. In addition, the large numbers of markers exhibiting small differences among sites may contribute little to Φ_ST_, but may allow assignment programs to distinguish populations. Simulation studies [47], [48] have suggested that assignment based methods perform relatively poorly in detecting genetic structure when populations are not highly differentiated, however these studies evaluated scenarios with relatively few loci (20–30 microsatellites) compared to the numbers of loci now available with recent techniques. Waser [49] has noted that assignment techniques that are based upon individual genotypes have many advantages over F_st_ based measures. Indeed the utility of AFLPs specifically for population assignments studies has been noted [29]. Finally, the sensitivity of assignment methods to detect population structure even when F_st_'s are not significant has been observed elsewhere [50]. Using 86 polymorphic AFLP markers we were able to correctly assign individuals back to the populations from which they were sampled with frequencies ranging from 89% to 100% (Table 5A). Even when assignments were limited to only those samples that had a ten-fold greater assignment probability than the next most likely assignment, 66% to 98% of the samples were correctly assigned to the depth from which they were collected (Table 5B). Within the four depths (separated by as little at 10 m) at LCI the AFLPOP analysis correctly assigned samples back to the correct depth with frequencies exceeding 66% (Table 5B). Similarly, correct assignment frequencies among depths at LSI were high, greater than 92% among the four depth ranges.

We also were able to differentiate among samples classified solely by depth, even when combining the samples from LCI and LSI. As was seen among depths within each collection site, AFLPs allow for the detection of potential genetic similarities by depth alone even when combining distant collection sites. Correct assignment frequencies ranged from 27% to 63% with the log likelihood ratio set to 1 (Table 6B). The lowest frequencies of successful assignment were among the shallow and medium depth populations. Differences among populations can also be seen in the DFA though there is some overlap among shallow and medium depth samples in the plot of canonical scores (Fig. 2). The presence of such genetic similarities among sites classified only by depth may indicate selection for some of the markers. Wilding *et al.* [51] found that 5% of the 306 AFLP markers used to assess gene exchange between morphs of the intertidal snail, *Littorina saxatilis*, exhibited greater differentiation (as measured by Φ_ST_ values) than expected based upon simulations suggesting selection on these markers or genes closely linked to them. Similarly, in this study the markers identified as contributing significantly to the discriminant function may reveal loci under local selection. Interestingly, the F_ST_ outlier analysis did not reveal any markers that were common to both sites. Rather, the analysis indicated that the two sites (LCI and LSI) may be experiencing different selection regimes. At LCI two of the markers identified show greater differentiation than would be expected of neutral loci suggesting positive selection. For both markers the differences appear to be greatest in comparing the shallower sites (S and M) with the deeper sites (D and VD, Fig. 3). This pattern of greater similarity among the two shallowest sites (S and M) as compared to the deeper sites (D and VD) was also found in the AFLPOP analyses (greater numbers of miss assignments) and the AMOVA (significant pairwise comparisons were between S/M vs D/VD). At both LSI and LCI markers were identified by the F_ST_ outlier analysis suggesting that balancing selection may also be important (Fig. 3).

The cause of genetic heterogeneity among depths for *Montastraea cavernosa* is unknown but may indicate that the populations sampled are sustained largely by local recruitment with low dispersal of larvae from natal populations. This seems improbable given the closeness (10′s of m) of the populations sampled within each site and the reproductive strategy of *M. cavernosa* as a broadcast spawner. Given that “adult” corals were sampled during this study and that the genetic structure of the populations is likely the result of multiple years' of recruitment, some sort of “sweepstakes recruitment” event is also unsatisfactory as an explanation for the observed results. Selection remains a possible explanation and is supported by the F_ST_ outlier analysis for at least LCI. Bongaerts *et al* [52] invoked selection to explain the strong genetic structure of the coral *Seriatpora hystrix* on the Great Barrier Reef over a 30 m depth range and later provided evidence that divergent selection probably led to local adaptation for these populations of *S. hystrix* [53]. Similarly, Prada and Hellberg [12] note that the divergence by depth seen in the Caribbean octocoral, *Eunicea flexuosa*, may be due to weak selection operating over the long pre-reproductive period of this species (15+ years) even in the face of what is presumed to be wide dispersal as evidenced by the little geographic differentiation among populations across the Caribbean. In this regard the genetic differentiation observed in this study could also be the result of strong selection on specific loci simultaneously with high levels of larval dispersal and gene flow that is unable to homogenize the effects of selection resulting in local adaptation [54]. It is interesting to note that the selection regimes operating at LCI and LSI may be different resulting in differentiation among populations at LCI and little differentiation at LSI. This assumes that the rates of immigration of larvae from a well-mixed, genetically heterogeneous, pool in the water column are similar at the two sites. Support for selection-mediated differentiation also comes from the depth dependent differentiation of *Symbiodinium* sp. phylotypes from the same colonies of *M. cavernosa* used in the AFLP analyses from LSI populations [55]. The break point (∼60 m) where unique phylotypes occur is the same depth where the host population genetics also differentiates significantly.

Despite the small sample sizes used here the utility of AFLPs to reveal genetic differences among individuals of this broadcast spawning coral on this very small geographic scale suggests that this marker system warrants strong reconsideration as a tool in population genomic analysis, particularly when sampling is constrained. The great numbers of polymorphic markers that can be assayed combined with the universality of the protocol are definitive strengths as a tool in assessing the genetic connectedness of coral reef organisms (see [56] for discussion of tradeoffs between number of loci and sample size for different markers).

Mesophotic coral reef fauna have received recent interest as a potential source of propagules for nearby shallow coral reef communities that are increasingly damaged due to natural and anthropogenic disturbances [9], [53]. However, evidence for population connectivity between shallow and deep reefs is equivocal (reviewed in [10]). In fact, the data for *Montastraea cavernosa* ([55], this study) support a model of depth-specific physiological changes across coral reef depth gradients in the Caribbean. These data are further supported by recent population genetic studies on the fauna of other Caribbean reefs [57], [58] and Pacific reefs [53], [59], [60]. Since mesophotic coral reefs are themselves the subject of recent changes in community structure [61], [62], the potential loss of genetically-unique deep reef populations argues for a management strategy independent of their role as a “seed-bank” [10].

## Methods

### Sample Collection

Coral samples were collected from three sites, LCI (19° 41′ N, 80° 03′ W), LSI (23° 47′ N, 76° 06′ W) and SS (24° 00′ N, 74° 40′ W). The distance between SS and LSI is ∼170 km. Distances between LCI and LSI and SS are 1640 and 1350 km respectively. Collection and CITES export permits for all coral samples were obtained from The Department of the Environment, Cayman Islands and The Department of Fisheries, Ministry of Agriculture and Marine Resources, Bahamas. Depth collections at these sites occurred at 3, 10, 15, 25, 30 50, 75, 60 and 90 m ±1 m. Samples were collected using both open and closed circuit mixed gas technical diving (LCI and LSI) as well as by using the Johnson-Sea-Link submersible (SS).

### Amplified Fragment Length Polymorphisms (AFLPs)

High molecular weight genomic DNA was isolated using the Wizard SV Genomic DNA Purification System, as per manufacturer's protocol (Promega, Madison WI) for animal tissues. Prior to DNA isolation, samples were macerated lightly in saturated EDTA-DMSO saline (SED) buffer and spun at 16,000× g for 5 min to pellet the zooxanthellae and debris from the homogenate. All DNA isolations were checked for zooxanthellae DNA contamination using stringent zooxanthellae specific PCR [32], [34]. All samples used in the analyses were confirmed to be free of detectable zooxanthellae DNA.

AFLPs, like other multi-locus techniques, generate many bands, some of which are sensitive to PCR reaction conditions. Here, we have processed samples from DNA isolation through the final selective PCR in large, random lots containing samples from all sites to distribute any experimental error that may have been introduced by reaction conditions in an unbiased fashion. In addition, all PCR reactions were done using one machine and the same thermal cycle profile. Finally, the final selective PCR step was repeated three times for each sample. A band was scored as present only if appeared in all three replicates.

AFLP analysis was performed following protocols based upon Vos *et al.* [63] and Suazo and Hall [64]. Briefly, DNA was digested and ligated to the adapters (*EcorRI* adapter: ^5′^-CTC GTA GAC TGC GTA CC-^3′^, ^3′^ -CAT CTG ACG CAT GGT TAA-^5′^ ; *Mse*I adapter: ^5′^-GAC GAT GAG TCC TGA G-^3′^, ^3′^-TA CTC AGG ACT CAT-^5′^) at 16°C overnight with 1 U of *Mse*I (New England Biolabs), 5 U *Eco*RI (Promega Corp), and 1 WeissU T_4_ DNA Ligase in 1X ligase buffer (0.1 mM ATP) with 0.5 M NaCl. Digested/ligated DNA fragments were diluted twenty-fold for the first pre-selective PCR amplification. Primers used in the “pre-selective amplification” were complementary to the adapters, with the addition of a single nucleotide - an “A” for the *Eco*RI adapters, and a “C” for *Mse*I adapters. Five µl of the diluted restriction-ligation reaction was added to 15 µl of PCR mix (200 µM each dNTP's, 1X PCR buffer, 3 mM MgCl_2_, 0.275 µM each primer and 0.5 U Master TAQ® [Eppendorf]). The pre-selective amplification program consisted of an initial cycle of 72°C for 2 min (to complete the ligation of the synthetic adapters), followed by 20 cycles of 94°C for 20 s, 56°C for 30 s, and 72°C for 2 min, with a final extension of 72°C for 20 min. The pre-selective PCR products were diluted ten-fold for use in the final “selective amplifications”. Primers used in the selective PCR had the same sequences as the pre-selective primers, with the addition of two additional nucleotides at the 3′ and a FAM tag on the 5′ end. Five µl of the diluted pre-selective PCR reaction products were added to 15 µl of the PCR mix (200 µM each dNTP's, 1X PCR buffer w/3 mM MgCl_2_, 0.275 µM *Eco*RI primer, 0.275 µM *Mse*I primer and 0.5 U Master TAQ® [Eppendorf]). The selective amplification program consisted of an initial cycle of 94°C for 2 min, 94°C for 20 s, 66°C for 30 s, and 72°C for 2 min. This was followed by 9 cycles of 94°C for 20 s, 66°C for 30 s (decreasing 1°C/cycle), and 72°C for 2 min and another 20 cycles of 94°C for 20 s, 56°C for 30 s, and 72°C for 2 min, finishing with 72°C for 20 min. Products for the selective PCR were run on an Amersham MegaBACE 1000 96 capillary sequencer at the University of Florida's Interdisciplinary Center for Biotechnology Research. Resulting electropherograms were analyzed using SoftGenetics GeneMarker® (ver 1.51) for bands ranging from 50 to 400 bp in size in 5 bp increments. AFLP markers were scored as present for an individual sample only if a band appeared in all three replicates runs. A total of 213 marker size classes were assessed (3 markers ×71 size classes from 50 to 400 bp at 5 bp increments). Of the 213 marker size classes only those markers with a minimum frequency of “band presence” greater than 5% (band present in at least 6 individuals of the 105 samples) were used in the final analysis.

Overall levels of genetic differentiation among sampled populations were assessed using a nested Analysis of Molecular Variance (AMOVA155, [65]) based upon the presence/absence data. For this analysis, a bootstrap of 5000 iterations was performed to estimate p values for population statistics - Φ_ST_. In addition a population assignment technique was used to assess the genetic structure of the samples collected from LSI and LCI. The program, AFLPOP, examines the AFLP banding patterns –presence/absence data – and calculates log-likelihood values for any individual's membership in a population. Each individual is allocated to the population showing the highest likelihood for that genotype [20], [66]. Assignments to populations were set to a log-likelihood threshold of either 0 or 1. With a log-likelihood threshold of 0 samples are simply assigned to the group with the highest probability. At an assignment threshold of 1 assignment of a sample to a population was not made unless the probability of the given assignment was 10 times more likely than the next most probable assignment. If this threshold is not met, the sample is assigned to the “none” category. It should be noted that a sample being assigned to the “none” category denotes that there are two or more populations with similar probabilities of assignment (i.e. less than a 10-fold difference) not that the sample could not be assigned to any population. Given the relatively large number of markers generated in this study compared to our sample sizes, the contribution of each individual sample to the group frequencies is expected to overestimate the level of correct assignments. In order to assess this effect we first performed an AFLPOP simulation analysis on the data with the individual samples randomly assigned to the six depths at LCI and LSI. In addition as noted above assignments were evaluated at the high stringency of a log likelihood threshold of 1. The purpose of this was to determine whether spurious, misleading patterns of population structure might be generated by chance alone given the large number of markers and the small populations sampled.

To assess the contributions of specific markers to the observed patterns two techniques were employed. Multiple discriminant function analysis (Statistica, ver 9.0 StatSoft Inc. Tulsa, OK) was performed to build a discriminant function model to assess the utility of AFLP data to differentiate among populations. A forward stepwise analysis was used to build a model that included only those markers that significantly contributed discrimination among groups. Discriminant canonical function scores can be visualized by plotting the individual scores by group membership. Finally, to identify markers that display unusually high of levels of genetic differentiation and therefore may be subject to selection in the LCI and LSI populations F_ST_ outlier analysis was conducted on for each data set with samples identified only by depth categories. The selection detection workbench, Mcheza [67], identifies loci with outlying values of F_ST_ identified in plots of F_ST_ versus expected heterozygosity [68] for dominant markers. Initial simulations were run for each dataset to estimate the mean neutral F_ST_ and identify outlying loci that may bias the estimation of the mean neutral F_ST_. A second run (100,000 simulations) using all loci was then conducted using the computed value for neutral F_ST_. Loci falling outside the 95% confidence intervals and with a false discovery rate (FDR) of 0.01 were considered putative candidates for loci under selection.
